# The Colour of the Night Sky

**DOI:** 10.3390/jimaging6090090

**Published:** 2020-09-05

**Authors:** Zoltán Kolláth, Dénes Száz, Kai Pong Tong, Kornél Kolláth

**Affiliations:** 1 Department of Physics, Eötvös Loránd University (ELTE) BDPK, 1053 Budapest, Hungary; szaz.denes@gmail.com (D.S.); tong.kai.pong@sek.elte.hu (K.P.T.); kollath.k@met.hu (K.K.); 2 Hungarian Meteorological Service, 1024 Budapest, Hungary

**Keywords:** light pollution, imaging radiometry, colorimetry, night sky colour, colour analysis, false colour enhancement

## Abstract

The measurement of night sky quality has become an important task in night sky conservation. Modern measurement techniques involve mainly a calibrated digital camera or a spectroradiometer. However, panchromatic devices are still prevalent to this day, even in the absence of determining the spectral information of the night sky. In the case of multispectral measurements, colour information is currently presented in multiple ways. One of the most frequently used metrics is correlated colour temperature (CCT), which is not without its limitation for the purpose of describing especially the colour of natural night sky. Moreover, visually displaying the colour of the night sky in a quantitatively meaningful way has not attracted sufficient attention in the community of astronomy and light pollution research—most photographs of the night sky are post-processed in a way for aesthetic attractiveness rather than accurate representation of the night sky. The spectrum of the natural night sky varies in a wide range depending on solar activity and atmospheric properties. The most noticeable variation in the visible range is the variation of the atomic emission lines, primarily the green oxygen and orange sodium emission. Based on the accepted models of night sky emission, we created a random spectral database which represents the possible range of night sky radiance distribution. We used this spectral database as a learning set, to create a colour transformation between different colour spaces. The spectral sensitivity of some digital cameras is also used to determine an optimal transformation matrix from camera defined coordinates to real colours. The theoretical predictions were extended with actual spectral measurements in order to test the models and check the local constituents of night sky radiance. Here, we present an extended modelling of night sky colour and recommendations of its consistent measurement, as well as methods of visualising the colour of night sky in a consistent way, namely using the false colour enhancement.

## 1. Introduction

In the last few decades, urbanisation and decreasing energy cost caused a dramatic increase in the extent of artificial lights at night (ALAN) [1]. Light pollution not only hinders astronomical observations, but also influences the natural behaviour of nocturnal animals, affecting foraging, reproduction, communication, and other critical behavioral patterns [2,3,4,5,6]. The mechanism of the effect of light pollution on a cellular level is also widely researched, revealing that increased illumination at night change the circadian rhythm and inhibits melatonin production causing adverse health issues in non-nocturnal species [7,8,9,10]. Humans are not an exception either, the latest studies show direct correlation between the colour of public lighting and tumour formation, e.g., in breast, prostate, and colorectal cancers [11]. In addition, overillumination implies a waste of energy, which, in turn, means unnecessary carbon emission (see for example [12] for method of using artificial light as a proxy for energy consumption). It is though not impossible to find and optimum between light requirements and minimal environmental impact, as we demonstrated in the areas of Zselic and Bükk starry sky parks in Hungary, where the whole lighting system of two settlements has been reconstructed in a pilot project based on a special new design of LED lamps [13]. To reliably measure light pollution, the knowledge on measurement techniques and the spectral monitoring of natural night sky is essential.

A frequently used device, the Sky Quality Meter (SQM), is a single-channel instrument that is able to measure sky brightness in and around the zenith in one channel of intensity with wide bandwidth. This method has several drawbacks for measuring light pollution. The SQM (e.g., [14]) possess an arbitrary filter that is generally not calibrated for any astronomical or photopic band, and it is excessively sensitive around the blue wavelengths. It does not use an SI-traceable unit, the intensity displayed based on the calibration of a stellar spectrum that has a different level at different wavelengths. Recently, Bará et al. [15] worked out a method for general measurement and an absolute radiometric calibration of SQM sensors and described the parameters that define the absolute radiance scale of AB magnitudes per square arcsecond, for their specific photometric bands. Nonetheless, measurements with an SQM lack the extra information obtained from a multi-channel method and, thus, are not directly comparable with those of other instruments, e.g., digital single lens reflex (DSLR) cameras or spectroradiometers. In addition, the angular sensitivity of the SQM’s optics is inconsistent across units, and the characterisation of one unit is, thus, possibly not applicable to another.

Multi-channel measurements provide not only intensity, but spectral information on the night sky, which enables a more complex sky quality analysis and light pollution measurements [16]. Digital cameras, such as DSLR and mirror-less (MILC) cameras that can save images in raw format, can be used to measure sky radiance after calibration, and various representations of the distribution of sky brightness (such as false colour images) are possible [17,18,19,20,21]. One of the methods for all-sky measurements is to use a fish-eye lens equipped on a digital camera, which can provide sufficient resolution in the area near the zenith and above the horizon. However, they are not appropriate for measurements around the horizon where the resolution and precision become poor, which is unfortunate, because these are the most interesting areas for light pollution research. This drawback can be solved without changing our device if we rotate the camera by 90 degrees and take two or multiple fish-eye images in the vertical plane [22]. The best way to achieve the highest precision and resolution at dark locations and under clear sky conditions is to use a robotic panorama head with a 24 mm or 35 mm rectilinear lens on a full-frame digital camera [23]. With this set-up, 28 individual images are enough to cover the whole sky and some of the ground and environment with high spatial resolution. The images are taken at different pointing direction, using 6–10 s of exposure time and ISO between 5000 and 10,000. Because of the short exposure time, the movement artefacts (like star trails) caused by the apparent rotation of the sky do not disturb the measurement. Astrometry-based corrections of the images can also be made during post-processing [23].

It is important to use an appropriately calibrated device for measurement, to have knowledge on the natural sky spectrum and to use an SI-traceable unit for dark sky characterization in order to reliably monitor light pollution. The most frequently used measurement devices are differently calibrated using different reference targets. Different research groups use different metrics and units and these units are not necessarily fully compatible with the standard definitions. For calibration, the application of a standard source or a separate device is the most common, the spectral characteristics of which differ from the spectral response of the measurement device. DSLR cameras are calibrated by astronomical or standard CIE photometry [24]. Previously, we have suggested a measurement method that is based on the calibration of DSLR cameras with known narrow-band light sources and a spectroradiometer with which the natural sky spectrum could be precisely determined [23]. We introduced the dark sky unit (dsu), which is a new SI-traceable unit (nW/m${}^{2}$/sr/nm) for measuring sky brightness and it can be separately determined for the three colour channels of the digital camera. The natural changes in the sky radiance, like airglow, the natural emission of the molecules, and atoms in the upper atmosphere, are also included in the measurement method.

For sky quality analysis, information regarding the natural dark sky is essential to be able to separate the spectral components that are caused by light pollution from those caused by natural phenomena. Even the clear natural night sky spectrum with no light pollution can be very different based on various factors such as geographical location, atmospheric and meteorological conditions, etc. (typical spectrum of night sky can be found in e.g., [25]. However, readers should be noted that apart from visible light spectrum it also covers from UV to microwave, which is beyond the scope of this article). The natural emission of the molecules and atoms in the upper atmosphere (airglow) is the most important dynamic component of night-sky radiance, especially at places with negligible light pollution. For example, oxygen primarily emits in the green spectral line, while sodium emits in the orange. Clouds and aerosols add another factor to sky brightness by reflecting light to the scattered light causing the radiation reaching the surface [26,27]. The contribution of clouds to sky brightness near urban areas are measured and explained in [28], the elevated level of brightness could be measured by all-sky photometry while using a fish-eye lens several kilometers from the city. Night sky brightness is often characterized by the correlated colour temperature (CCT). Under clear sky, due to the Milky Way, the CCT in the zenith can reach 4700 K which decreases towards the horizon. Under overcast sky the maximum CCT is around 3000 K [27]. Although CCT representation is useful to distinguish clear sky from cloudy sky and to detect various sources of light pollution, it does not characterize well the real colour of the sky. The colour channels of DSLR cameras are usually calibrated based on the CIE 1931 colour space and based on that the CIE XYZ colour representation describe the sky colour better than the CCT value. Another advantage of CIE XYZ is that it can be easily converted to other colour spaces (e.g., Adobe RGB), where light polluting sources are easier to separate from the natural sky brightness.

In this paper, we present a method to determine the real colour of the night sky while using all-sky images taken by a digital DSLR or MILC camera based on the method in [23]. In our previous paper, we provided recommendations to an SI traceable metric for the measurement of night sky radiance. The introduced camera-based band-averaged radiance can be measured in nW/m${}^{2}$/sr/nW units abbreviated as dsu (Dark Sky Unit); it is a natural choice as the non-polluted sky has radiances in the order of 2–3 dsu. In this paper, we extend this recommendation, defining a colour space that is based on normalised band-averaged radiance values and the possible conversion between different colour spaces. Here, we show how different the various night skies (light-polluted and natural) can be in different colour spaces.

## 2. Materials and Methods

The spectral measurements were obtained with a Konica Minolta CS–2000 Spectroradiometer. The device was used with the maximum possible aperture. The exposition is set to automatic resulting in the maximum exposure time (2 min.). The sensitivity of this spectroradiometer makes it possible to obtain sky spectra, even at natural sky conditions. Usually, we take 5–10 spectra at the same location and direction to improve the signal-to-noise ratio of the measurement.

All of the all-sky measurements presented in this paper were acquired by Sony ILCE 7SII cameras equipped with Samyang 24 mm T1.5 VDSLR ED AS IF UMC II lens. The camera was attached to a robotic panorama head (GigaPan EPIC Pro). At a given location, we took 28 or 35 images with the motorized head to cover the whole sky and the portion of terrain close to the horizon with high resolution. The standard exposure setup for the measurements: ISO: 6400, exposure time: 6 s, F/1.4 (T1.5) aperture.

The individual images are processed to calibrated radiance distributions of the sky in dsu units. Then the images were stitched to each other to generate hemispheric images in spherical projection. All of the image processing besides the stitching of the images was performed by our DiCaLum library written in GNU Octave.

The synthetic data used in this paper originate from the web-page of the Advanced Cerro Paranal Sky Model [29]. We separated the different components of the model spectra by cutting the different spectral ranges and also by fitting the standard emission profiles matching the bandwidth of our spectroradiometer. Our measurements by the spectroradiometer provided additional sky spectra with (twilight and zodiacal light). The details of these fits are presented in the next section.

## 3. The Spectral Information of the Night Sky Colour

The spectrum of the night sky contains the fingerprints of the different light sources. Therefore spectroradiometry provides the most complete information about sky radiance.

**Figure 1:**
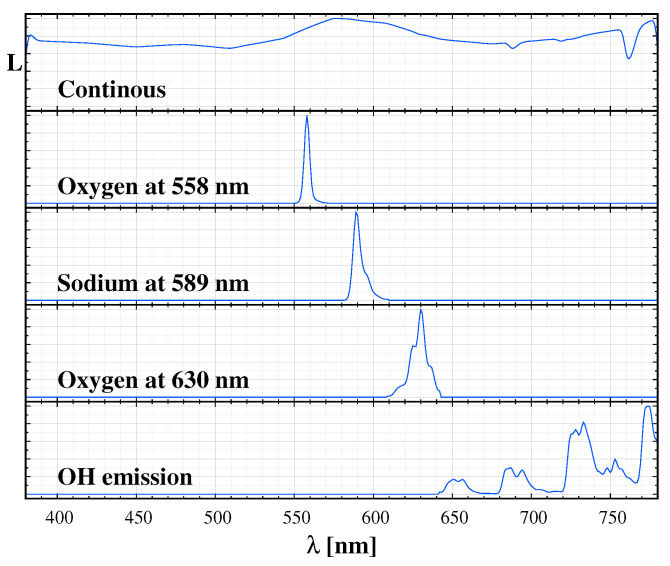
Components of the natural sky spectrum used to fit the observations. From top to bottom: continuous component, 558 nm (green) oxygen emission, 589 nm sodium emission, 630 nm (red) oxygen emission, OH emission.

**Figure 2:**
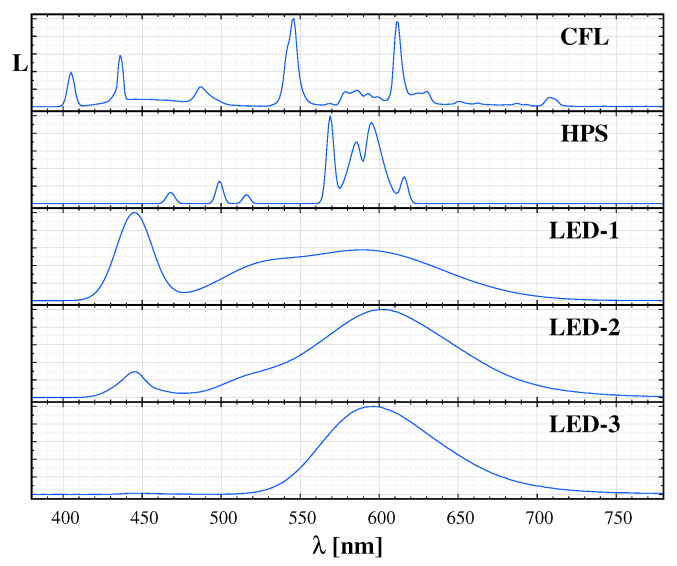
Samples of the used light source spectrum database to fit night time spectra. CFL: compact fluorescent lamp; HPS: high pressure sodium, LED-1: cold white LED; LED-2: warm white LED; LED3: phosphor converted amber LED.

We performed an extended spectral survey in the Zselic Dark Sky Park, Hungary. In [23], we already demonstrated that the spectrum can be perfectly fitted by the natural sky model and the spectrum of the light sources in the neighbouring settlements. A fit with the components of the natural sky spectrum and the typical artificial light sources (blue and orange components of white LED, compact fluorescent lamp, and high-pressure sodium lamp) model the observed spectrum well. We created a spectrum catalogue based on the spectra of the common public lighting sources and the fit to the local measurements based on the “Advanced Cerro Paranal Sky Model” [29]. Figure 1 displays the major components of the natural sky radiance and Figure 2 shows a sample of the typical sources in the Zselic region. The continuous part, which consists of the zodiacal light, scattered starlight, and the residual continuum of the airglow, is an approximately flat curve at 2 nW/m${}^{2}$/sr/nm.

As a starting point, we selected a low and high standard value of the natural sky radiance based of the Paranal Sky model corresponding to 60 sfu and 120 sfu monthly averaged solar radio flux. In our standard measurements survey, we use dark sky unit (dsu) as a metric for sky glow measurement. Thus, we parameterized the spectral sequence ${\bar{L}}_{G}$ with this unit. Table 1 lists the main components of the natural sky radiance and our accepted radiance limits for the standard low (L) and high (H) values. Please note that, especially the oxygen and sodium emission, can significantly exceed even the normal high values. We refer to this range of radiance as the standard sky. We selected random sets of parameters uniformly distributed between the ${\bar{L}}_{G}^{\left(L\right)}$ [dsu] and ${\bar{L}}_{G}^{\left(H\right)}$ [dsu] limits.

**Figure 3:**
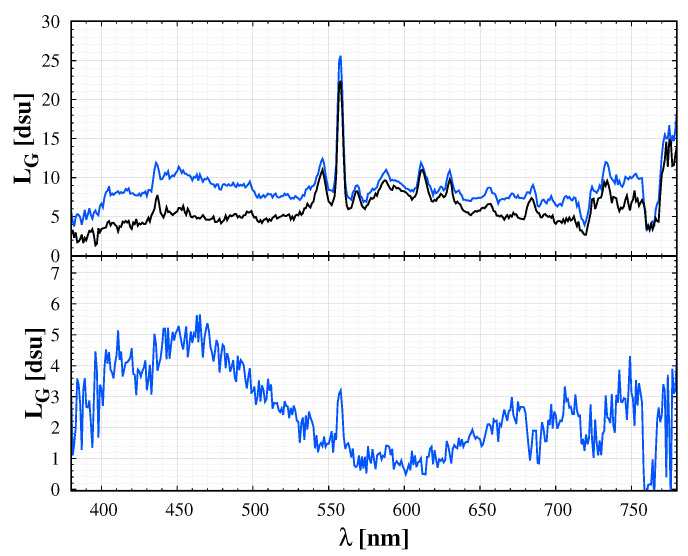
**Top panel**: the spectrum of the sky at a fixed location during astronomical night (black curve) and during twilight (blue curve). The lower panel displays the difference of the two above curves, the twilight component. Components of the natural sky spectrum used to fit the observations.

After extreme solar activity, like high extreme UV radiation, the level of airglow can increase by a factor of 5–10. Airglow emission is also effected by atmospheric conditions, like gravity waves. Thus, the real airglow level has a larger variability. In addition, here we demonstrate how differential spectroscopy help in separating faint components of night sky spectrum. Figure 3 shows that differentiating sky spectrum for different times during a night removes the ALAN components and most of the natural airglow. The resulting curve is a good approximation of the twilight components. The colour of the twilight sky is frequently misinterpreted. The Rayleigh-scattering alone cannot—give a bluish tint. The ozone absorption in the upper atmosphere is the crucial mechanism—the dip in the whole central part of the visible spectrum is the Chappuis absorption band of ozone [30]. The CIE colour coordinates of the twilight component are x,y = 0.22,0.22, indicating a clear blue colour. However, it is mixed with the other components of the sky; therefore, it is shifted to the orange direction. Please note that here we provided the twilight spectrum for completeness of night sky information at first. In the following sections, we first concentrate on the sky colour during the astronomical night. Therefore the twilight spectrum is not included in these calculations, as for light pollution measurement we restrict the observing time for the astronomical night. However, for a general image processing scenario, we later extend the learning set by a twilight component.

**Figure 4:**
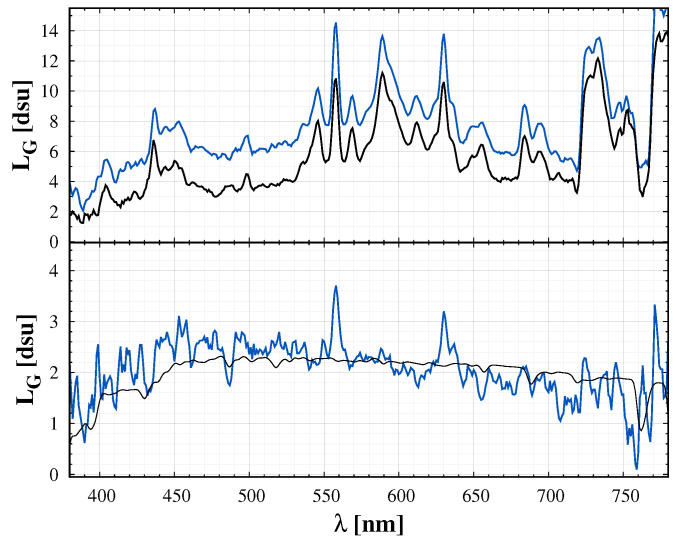
**Top**: Night sky spectrum with the zodiacal light (blue) and at a close sky location (black). The features of the light polluted sky is clearly visible, but the difference of the two spectra (lower panel, blue curve) emphasize only the zodiacal light and some components from airglow. The lower panel also displays the model of zodiacal light with black curve (Advanced Cerro Paranal Sky Model, see in Section 4).

Figure 4 shows another demonstration of differential spectroradiometry. Here, the difference between the spectra taken at different directions removes the ALAN and natural components and the remaining part provides an estimate of the zodiacal light.

## 4. Sky Colour Modelling

We generated a sequence of spectra with different combination of its components in order to demonstrate the effect of different natural phenomena and the light pollution on the colour of the night sky the observable quantities. The spectra of the natural components are based on the “Advanced Cerro Paranal Sky Model” (https://www.eso.org/sci/software/pipelines/skytools/skymodel) [29]. We use two extreme models. The low radiance model is calculated at the ecliptic pole, with “Monthly Averaged Solar Radio Flux” of 60 sfu during the middle third of the night in December/January. The effect of moonlight is neglected. We calculated the high radiance model with doubled (120 sfu) radio flux, for April/May conditions. The other parameters are the same. The seasonal change and the increased solar activity provide a significant increase in the natural background (e.g., ${\bar{L}}_{G}$ increases from 1.7 dsu to 3.1 dsu). In the table, we present the theoretical continuous and the emission line contribution separately for the extreme cases.

A model spectrum can be calculated based on the spectral components that are provided in Table 1 and in Figure 1 and Figure 2, with any relative ratio of the different components. Subsequently, we followed a standard procedure to calculate the CIE xy colours for a given spectral distribution S(λ), i.e., We integrated the products of S(λ) and the CIE 1931 colour matching functions to obtain the XYZ tristimulus values, and then we got the colour coordinates in the CIE 1931 xy chromaticity diagram. It would also be possible to generate the colour coordinates in the CIE L${}^{*}$a${}^{*}$b${}^{*}$ colour space, but, according to our experience, it does not provide additional information on sky quality and colour. However, we apply our results to digital camera measurements; thus, it is straightforward to use camera based colours. The measurements provide the RGB band-averaged radiances: ${\bar{L}}_{R}$, ${\bar{L}}_{G}$ and ${\bar{L}}_{B}$. From this triplet, we define the camera-based colour coordinates as $\ell_{R}={\bar{L}}_{R}/\left({\bar{L}}_{R}+{\bar{L}}_{G}+{\bar{L}}_{B}\right)$ and $\ell_{G}={\bar{L}}_{G}/\left({\bar{L}}_{R}+{\bar{L}}_{G}+{\bar{L}}_{B}\right)$. Then the triplet (${\bar{L}}_{G}$, $\ell_{R}$, $\ell_{G}$) fully describes the measurement for a given pixel. These values can be calculated from synthetic spectra similarly to the derivation of CIE xy coordinates, but this time the camera RGB spectral sensitivity curves are integrated with a given spectrum.

**Figure 5:**
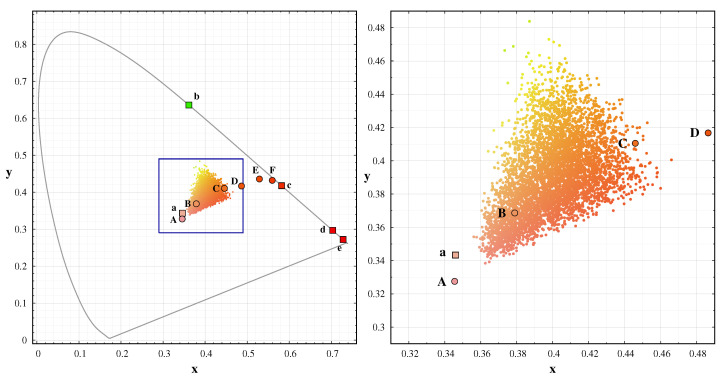
CIE 1931 colour diagram of the model spectra (small dots) used for the colour conversion definition. The rectangle shown in the **left** figure is enlarged in the right box. The large symbols represents the spectral database used for the models: A: 4900 K LED; B CFL; C: 2900 K LED; D: 2400 K LED, E: HPS; F: Amber LED; a: continuous natural component, b: green oxygen emission, c: sodium emission, d: red oxygen emission, e: OH emission.

Figure 5 displays the CIE 1931 colour coordinates of the spectra in the database. Contrary to the usual impression of bluish night sky colour, in reality the sky is in the orange to green range. Blue colour only appears during twilight hours, due to ozone absorption.

**Figure 6:**
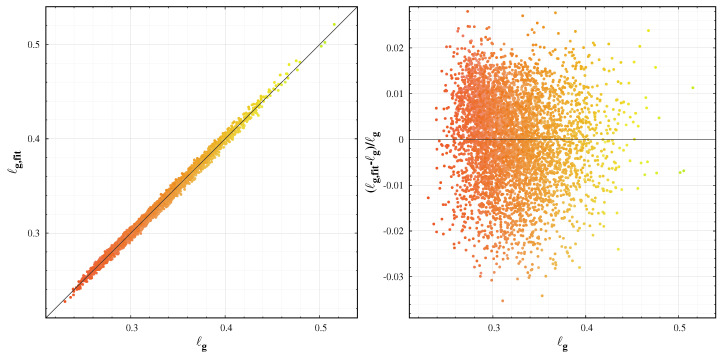
The quality of the colour conversion for the green channel. **Left box**: the fitted versus original $\ell_{g}$ coordinates, **right box**: the error of the fit. The dots represent the actual colours.

Figure 6 shows the quality of the fit of the colour transformation for the green channel of the data presented in Figure 5. For the given set of data, the error of the green channel conversion is within 2%. For the blue channel, we found a similar error, however the red colour has more variability and the error of the fit reaches 10%. The better precision in the blue and green channels suggests that these two colours are better choices for representing the colour of the sky.

## 5. Conversion between Colour Spaces

We used our spectral database as a learning set, and fitted the colour transformation matrix. The real RGB colours (in standard sRGB colour-space) were calculated from the CIE xy coordinates by the published colour matrix (the CIE xy coordinates are obtained in the standard CIE procedure from the spectra).

Subsequently, we used the camera RGB colour response to calculate the corresponding dsu colour values. We normalized the colours to ${\bar{L}}_{R}+{\bar{L}}_{G}+{\bar{L}}_{B}=1$. We used this training set to fit the colour transformation matrix from the dsu based colours to the sRGB colour space.

The 3 × 3 colour correction matrix provides the transformation from the camera ${\bar{L}}_{\mathrm{RGB}}$ values to corrected colours:

(1) $$\begin{array}{cc} R= & 1.38\;{\bar{L}}_{R}+0.02\;{\bar{L}}_{G}+0.18\;{\bar{L}}_{B} \end{array}$$

(2) $$\begin{array}{cc} G= & -0.32\;{\bar{L}}_{R}+1.40\;{\bar{L}}_{G}-0.19\;{\bar{L}}_{B} \end{array}$$

(3) $$\begin{array}{cc} B= & -0.06\;{\bar{L}}_{R}-0.42\;{\bar{L}}_{G}+1.37\;{\bar{L}}_{B} \end{array}$$

Please note that this transformation is not a unique one, it depends on the learning set of different spectra. However, the final colours do not change significantly when the learning set is modified. We use this transformation throughout this paper.

**Figure 7:**
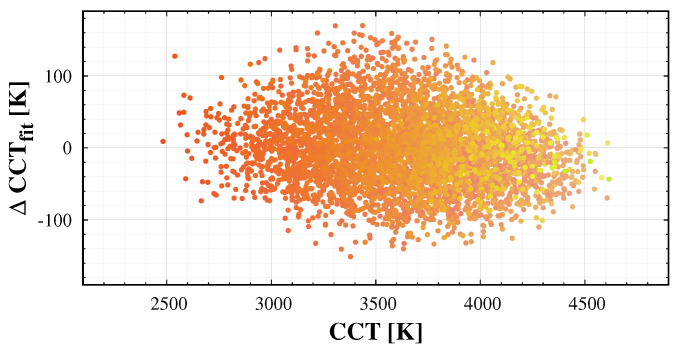
The error of the quadratic fit of correlated colour temperature (CCT) to the camera band-averaged spectral radiance.

In addition to the standard colour conversion, it is possible to fit the correlated colour temperature (CCT) based on the training spectrum data set. However, one should use at least a quadratic fit to the colours, to get a satisfactory result. Additionally, the error of the fit is relatively large, as demonstrated in Figure 7. In addition, spectra with similar camera based colours may have different CCT values. Therefore, we recommend to use the colour coordinates representing the images instead of the approximated CCT of the sky.

## 6. Application to Image Processing

Astrophotographers usually set the white balance manually to achieve bluish-gray night sky colour. Another option to shift the hue of the image is to set the colour temperature of the white balance setting to lower values (3000–4000 K). This setup automatically produces neutral or blue colors. However, the right colour of the sky is not neutral or bluish, as was demonstrated in the previous sections. Based on the colour transformation defined in Section 5, we can convert the raw camera images to dsu scale and then produce the images in colours close to the real spectral radiance of the sky.

Here, we present some basic case studies on the application of our colour transformations. The most straightforward one is the image processing of the natural sky with no light pollution. This provides the basis for further studies where some of the emission sources (natural or artificial) exceed the others. It gives a possible reference point. The next example is a location with low light pollution (dark sky park in Hungary); it demonstrates the effect of light pollution on colours. The third demonstration is the processing of aurora images. Here, the correct colour processing is essential, since the source of the emission is then predictable from the colours. In contrary, the erroneous processing of the images may predict incorrect physical process behind the emission. In a forthcoming paper, we analyse additional night sky scenarios, for example, observations with intensive airglow in the oxygen or sodium lines. We are performing a long term light pollution survey in Hungarian national parks and some other locations—when a sufficient amount of data will be collected, we will analyse these data with the same methodology.

### 6.1. General Night Sky Images

**Figure 8:**
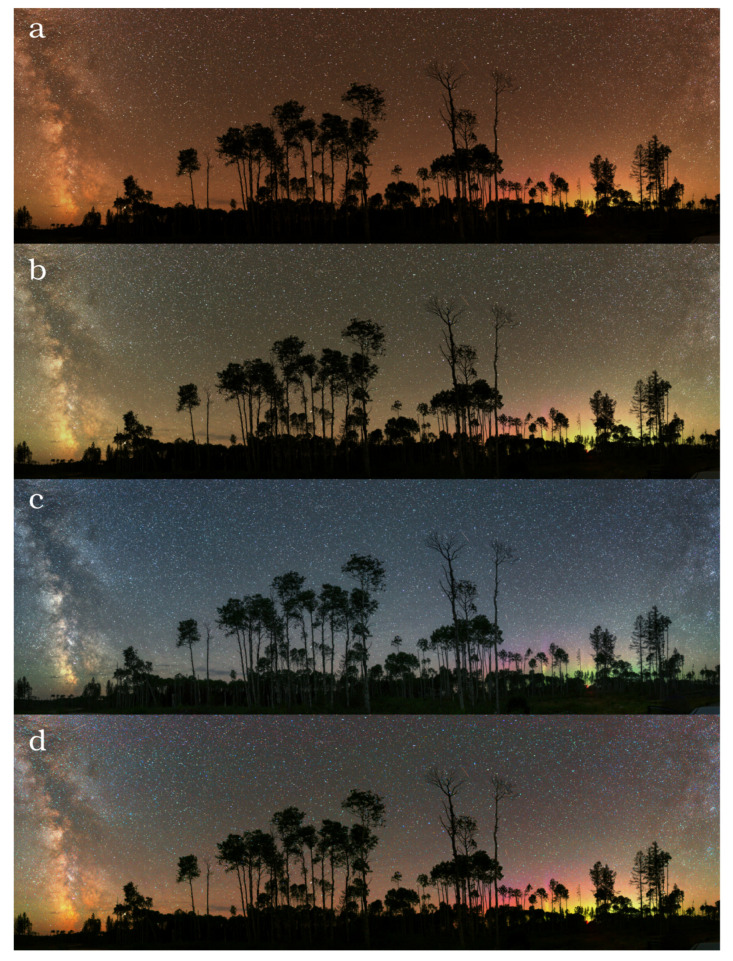
Different representations of the same photo taken at a light pollution free location in Ontario, Canada (the coordinates are presented in the text). (**a**) real colours (**b**) 3800 K white balance setup, (**c**) manual additional processing, and (**d**) false colour enhancement.

**Figure 9:**
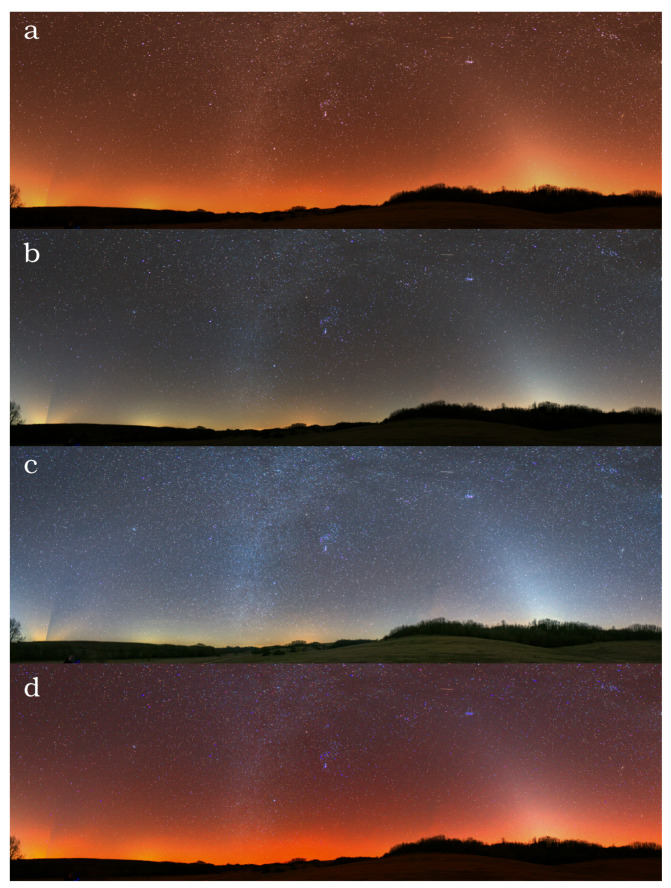
Different representations of the same photo taken at a moderately light polluted location at the Zselic Landscape Protection Area, Hungary. (**a**) real colours (**b**) 3800 K white balance setup, (**c**) manual additional processing, (**d**) false colour enhancement.

We selected a location with no light pollution and minimal airglow activity (some aurora activity close to the horizon) in Canada (coordinates: 48.2259${}^{\circ }$, –82.4393${}^{\circ }$) on 9 March 2019 at 02:30 UT. For comparison, we included an image from a mildly light polluted location (Zselic Starry Sky Park, Hungary (coordinates: 46.2903${}^{\circ }$, 17.6840${}^{\circ }$) Figure 8 and Figure 9 display four different versions of the same image, taken at a light pollution free and a moderately light polluted location. From top to bottom, the following are shown: (a) the real colour image calculated from the dsu conversion of the raw camera image, (b) image with white balance set to 3800 K in order to get neutral colours of the sky, (c) image with manual post-processing often applied by photographers to further enhance bluish glow of the sky and the stars, and (d) image with false colour enhancement (FCE) to visualize light pollution better. We defined the FCE colour conversion matrix with the following procedure: starting from the CIE x–y coordinates, we defined a shifted x–y colour pair for all the spectra of the learning set. The shifted x–y pairs are defined by the following constraints: the mean natural sky is shifted to the white point of the sRGB colour space; distance from the white point is doubled. The following conversion satisfies the above criteria well:

(4) $$\begin{array}{c} x_{FCE}=0.42+2\;\left(x-0.42\right) \end{array}$$

(5) $$\begin{array}{c} y_{FCE}=0.39+2\;\left(y-0.39\right) \end{array}$$

The above formula is not a unique one. We tested several alternatives and selected the one with optimal differences in colour representations of different emission scenarios we have. The first idea was to use for projection point the centre of the triangle in the $\ell_{R}-\ell_{G}$ plots. However, it resulted in an orange scent for most of the possible colours. Subsequently, we selected $x_{c}=0.42$ and $y_{c}=0.39$ as the central projection point based in a manual trial and error procedure. We note that with an extended database of imaging sky radiance observations, a different and more optimal transformation can be derived. To obtain the extended (FCE) sRGB coordinates from camera data, we estimated the conversion matrix by applying the standard CIE x-y to sRGB conversion matrix. Then we fitted the final dsu colour ($\ell_{R}$, $\ell_{G}$, $\ell_{B}$) to sRGB transformation on the learning set. For the natural sky, it provides an image that is close to the astrophotographer approach, but by further colour enhancement. It should be noted that it is a false colour representation of the night sky, but with a well defined procedure. In contrast to FCE, the manual colour enhancement used by astrophotographers does not have an exact definition and it varies from image to image.

### 6.2. Aurora and Related Events

In 2016, a new upper atmospheric phenomenon, named STEVE (Strong Thermal Emission Velocity Enhancement), caused big surprise to auroral researchers. On digital photographs, STEVE looked like an elongated auroral arc. Although its colours resembles to aurora, researchers assume that the origin of the two phenomena is not related [31]. In the lower atmospheric region, another phenomenon, the Picket Fence, produced similar colourful jet, but there is no evidence of its physical relation to STEVE. For the first time, Gillies et al. [32] measured the spectrum of the two phenomena while using a Transition Region Explorer Spectrograph (TREx), which is a very sensitive imaging spectrograph sensitive between the 400 and 800 nm region. TREx is usually used for airglow and aurora measurements on the night sky under very low luminosities. The spectral mesurements of STEVE proved that it is basically different from the spectrum of aurora or Picket Fence [32].

**Figure 10:**
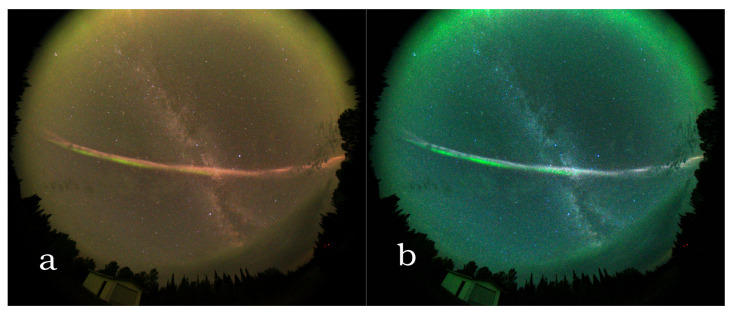
Different representations of the same photo taken of Strong Thermal Emission Velocity Enhancement (STEVE) at a light pollution free location. (**a**) real colours (**b**) 3800 K white balance with additional processing.

In photographs, STEVE often has deep purple or reddish colour. In Figure 10, different colour representations of STEVE are shown in a light pollution free area (Ontario, Canada, coordinates 49.5681${}^{\circ }$, –81.4215${}^{\circ }$). In real colour or dsu based colour representation, the phenomenon is not as bright and purple as photographers usually illustrate applying different colour enhancements, although the structures become also less visible. However, the real colour representation clearly shows the coexistence of red and green oxygen emission.

## 7. Discussion

Hollywood movies and astrophotographs usually visualize the night sky in a dark bluish colour that adds a spectacular glare to the apparent airglow. The reason for the former is that these movies are captured in daylight and the night effect is added during the post-processing of the cinema, astrophotos, however, are manually enhanced to obtain more vivid colours. Actually, our colour (photopic) vision is not working under very low luminance at night; thus humans cannot see the real colour of the night sky. In this paper, we demonstrated a method with which we could visualize the real night sky colour based on digital camera measurements and with the same colour conversion technique, we defined FCE in order to highlight the sources of light pollution and any enhanced natural sources. It is clear that the usually applied astrophotography style of image processing provides a false colour representation of night sky colour. Besides, it is an arbitrary procedure, with subjective steps. The FCE method provides a repeatable, measurement-based enhancement of night sky images. Therefore, when the image processing is performed not only for aesthetic purposes, but for scientific analysis of the sky imagery, FCE provides an optimal solution.

**Figure 11:**
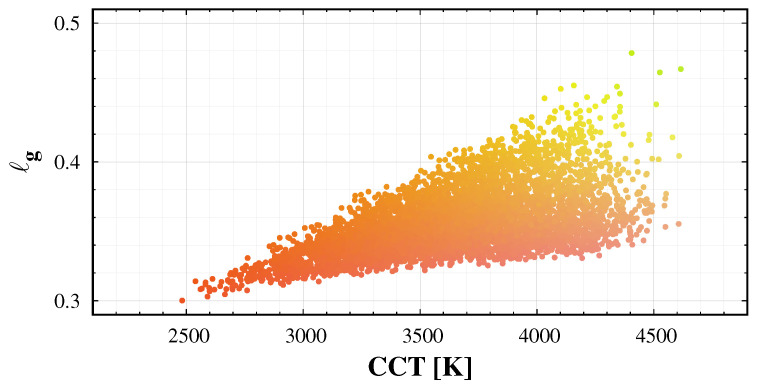
Correlated colour temperature (CCT)-colour diagram of the learning set: $\ell_{G}$ as a function of CCT. The dots represent the actual colours.

In the sky glow measurement literature, the colour of the night sky is usually characterized by the correlated colour temperature (CCT) of the sky. Figure 11 displays the normalized green colour ($\ell_{g}$), as a function of CCT. Especially in the case of high CCT, different colours are possible with the same calculated CCT. A single parameter cannot specify the colour of the sky. In addition, please note that the green colours are not compatible with black-body radiation. Thus, we recommend using two colour coordinates instead of correlated colour temperature when representing the colour of the night sky.

**Figure 12:**
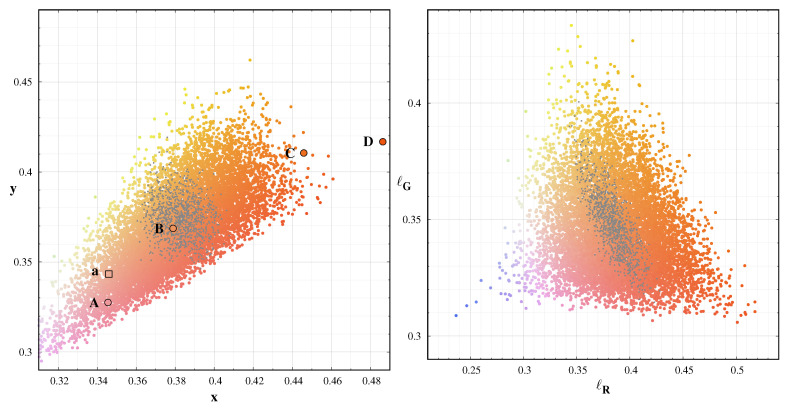
Colour-colour diagrams for an extended dataset. Left: CIE x-y colours, Right: $\ell_{R}-\ell_{G}$ colour space. The dots represent the actual colours; grey dots: natural spectra only.

We extended the range of possible spectra with the twilight spectrum of the sky. Besides, we increased the possible range of artificial light by a factor of two. Figure 12 displays the colour-colour diagrams for this set. The comparison of the CIE x-y and the dsu based ($\ell_{R}$–$\ell_{G}$) diagram demonstrated, the camera-based colours with the use of band-averaged spectral radiance is equally useful to represent sky colours. Thus, we can eliminate colour transformation errors with SI traceable colour coordinates.

The well-defined triangle structure in the $\ell_{R}$–$\ell_{G}$ diagram is the consequence that the visual radiation of the night sky is composed of three main components: the bluish twilight spectrum of the solar radiation, the green oxygen airglow, and a combination of different orange and red spectra. This third component includes the sodium and red oxygen airglow and most of the light pollution sources. These three primaries span the possible colour range of the night sky colours; thus shifts from the central “mean” natural colours of the sky indicates and excess in any of these components.

Continuous night sky quality surveys with comparable devices will provide a data mine for atmospheric studies, including the effect of natural airglow variations and the impact of aerosols on sky brightness and colour. A network of all-sky measurement cameras is under development in Hungary. In parallel, a portable system with higher resolution is used to add additional data coverage to the fixed system. The colour processing system that is defined in this paper will be a fundamental tool in the data processing pipeline.

## 8. Conclusions

Based on realistic models of night sky spectra and spectral observations, we determined the possible range of colours of natural sky emission during moonless astronomical night. The most possible natural sky colour lays in the following regions in different colour schemes:

- in CIE L*a*b*: −5<a*<20 and 15<b*<35.
- in CIE xyY: 0.36<x<0.41 and 0.35<y<0.41.
- in camera $L_{G}\ell_{R}\ell_{G}$: $0.34<\ell_{R}<0.44$ and $0.31<\ell_{G}<0.39$

We defined colour transformations from camera-based band-averaged radiances to different colour schemes while using a learning set defined by the possible spectra of the night sky. In addition to real colour representation, which provides the correct colour of the sky, we present a false colour enhancement (FCE) colours. The FCE representation cannot be used to reproduce the real colours of the night sky, but it provides an image representation of astrophotography which separates the different physical scenarios behind the emission of the sky. This method can be applied to light pollution surveys to help the interpretation of measurements. We provide such applications in a forthcoming paper.

## Figures and Tables

**Table 1:** Components of the natural sky radiance.

Source	${\bar{L}}_{G}^{\left(L\right)}$ [dsu]	${\bar{L}}_{G}^{\left(H\right)}$ [dsu]
Scattered starlight and zodiacal light	0.93	1.28
Airglow/residual continuum	0.55	1.10
558 nm oxygen emission	0.13	0.53
589 nm sodium emission	0.05	0.12
630 nm oxygen emission	0.02	0.07

## Abbreviations

The following abbreviations are used in this manuscript:

CCT	Correlated colour temperature
dsu	Dark sky unit
FCE	False Colour Enhancement
sfu	Solar flux unit
STEVE	Strong Thermal Emission Velocity Enhancement

## References

[1] Kyba C.C.M., Kuester T., Sánchez de Miguel A., Baugh K., Jechow A., Hölker F., Bennie J., Elvidge C., Gaston K., Guanter L. (2017). Artificially lit surface of Earth at night increasing in radiance and extent. Sci. Adv..

[2] Longcore T., Rich C. (2004). Ecological light pollution. Front. Ecol. Environ..

[3] Gaston K.J., Duffy J.P., Gaston S., Bennie J., Davies T.W. (2014). Human alteration of natural light cycles: Causes and ecological consequences. Oecologia.

[4] Gaston K.J., Gaston S., Bennie J., Hopkins J. (2015). Benefits and costs of artificial nighttime lighting of the environment. Environ. Rev..

[5] Grubisic M., van Grunsven R.H.A., Manfrin A., Monaghan M.T., Hölker F. (2018). A transition to white LED increases ecological impacts of nocturnal illumination on aquatic primary producers in a lowland agricultural drainage ditch. Environ. Pollut..

[6] Hölker F., Wolter C., Perkin E.K., Tockner K. (2010). Light pollution as a biodiversity threat. Trends Ecol. Evol..

[7] Aubé M., Roby J., Kocifaj M. (2013). Evaluating potential spectral impacts of various artificial lights on melatonin suppression, photosynthesis, and star visibility. PLoS ONE.

[8] Sanders D., Kehoe R., Tiley K., Bennie J., Cruse D., Davies T.W., Frank van Veen F.J., Gaston K.J. (2015). Artificial nighttime light changes aphid-parasitoid population dynamics. Sci. Rep..

[9] Gaston K.J., Bennie J., Davies T.W., Hopkins J. (2013). The ecological impacts of nighttime light pollution: A mechanistic appraisal. Biol. Rev. Camb. Philos. Soc..

[10] Kupprat F., Hölker F., Kloas W. (2020). Can skyglow reduce nocturnal melatonin concentrations in Eurasian perch?. Environ. Pollut..

[11] Garcia-Saenz A., Sánchez de Miguel A., Espinosa A., Valentin A., Aragonés N., Llorca J., Amiano P., Martín Sánchez V., Guevara M., Capelo R. (2018). Evaluating the Association between Artificial Light-at-Night Exposure and Breast and Prostate Cancer Risk in Spain (MCC-Spain Study). Environ. Health Perspect..

[12] de Miguel A.S., Zamorano J., Castaño J.G., Pascual S. (2014). Evolution of the energy consumed by street lighting in Spain estimated with DMSP-OLS data. J. Quant. Spectrosc. Radiat. Transf..

[13] Száz D., Kolláth Z., Szabó F., Csuti P. (2019). Living Environmental Laboratory for Lighting: Reduction of Light Pollution at Hungarian Settlements. Int. J. Sustain. Light..

[14] Cinzano P. (2005). Night sky photometry with sky quality meter. ISTIL Int. Rep..

[15] Bará S., Tapia C., Zamorano J. (2019). Absolute Radiometric Calibration of TESS-W and SQM Night Sky Brightness Sensors. Sensors.

[16] Hänel A., Posch T., Ribas S.J., Aubé M., Duriscoe D., Jechow A., Kollath Z., Lolkema D.E., Moore C., Schmidt N. (2018). Measuring night sky brightness: Methods and challenges. J. Quant. Spectrosc. Radiat. Transf..

[17] Kolláth Z. (2010). Measuring and modelling light pollution at the Zselic Starry Sky Park. J. Phys. Conf. Ser..

[18] Kolláth Z., Dömény A., Kolláth K., Nagy B. (2016). Qualifying lighting remodelling in a Hungarian city based on light pollution effects. J. Quant. Spectrosc. Radiat. Transf..

[19] Kolláth Z., Dömény A. (2017). Night sky quality monitoring in existing and planned dark sky parks by digital cameras. Int. J. Sustain. Light..

[20] Jechow A., Hölker F., Kolláth Z., Gessner M.O., Kyba C.C.M. (2016). Evaluating the summer night sky brightness at a research field site on Lake Stechlin in northeastern Germany. J. Quant. Spectrosc. Radiat. Transf..

[21] Jechow A., Ribas S.J., Domingo R.C., Hölker F., Kolláth Z., Kyba C.C.M. (2018). Tracking the dynamics of skyglow with differential photometry using a digital camera with fisheye lens. J. Quant. Spectrosc. Radiat. Transf..

[22] Jechow A., Kyba C.C.M., Hölker F. (2019). Beyond All-Sky: Assessing Ecological Light Pollution Using Multi-Spectral Full-Sphere Fisheye Lens Imaging. J. Imaging.

[23] Kolláth Z., Cool A., Jechow A., Kolláth K., Száz D., Tong K.P. (2020). Introducing the dark sky unit for multi-spectral measurement of the night sky quality with commercial digital cameras. J. Quant. Spectrosc. Radiat. Transf..

[24] CIE ISO23539:2005(E)/CIE S 010/E:2004 (2005). Photometry—The CIE System of Physical Photometry.

[25] Leinert C., Bowyer S., Haikala L., Hanner M., Hauser M., Levasseur-Regourd A.C., Mann I., Mattila K., Reach W., Schlosser W. (1998). The 1997 reference of diffuse night sky brightness. Astron. Astrophys. Suppl. Ser..

[26] Aubé M., Kocifaj M., Zamorano J., Solano Lamphar H.A., Sanchez de Miguel A. (2016). The spectral amplification effect of clouds to the night sky radiance in Madrid. J. Quant. Spectrosc. Radiat. Transf..

[27] Jechow A., Hölker F., Kyba C.C.M. (2019). Using all-sky differential photometry to investigate how nocturnal clouds darken the night sky in rural areas. Sci. Rep..

[28] Jechow A., Kolláth Z., Ribas S.J., Spoelstra H., Hölker F., Kyba C.C.M. (2017). Imaging and mapping the impact of clouds on skyglow with all-sky photometry. Sci. Rep..

[29] Noll S., Kausch W., Barden M., Jones A.M., Szyszka C., Kimeswenger S., Vinther J. (2012). An atmospheric radiation model for Cerro Paranal: I. The optical spectral range. Astron. Astrophys..

[30] Gadsden M. (1957). The colour of the zenith twilight sky: Absorption due to ozone. J. Atmos. Terr. Phys..

[31] MacDonald E.A., Donovan E., Nishimura Y., Case N.A., Gillies D.M., Gallardo-Lacourt B., Archer W.E., Spanswick E.L., Bourassa N., Connors M. (2018). New science in plain sight: Citizen scientists lead to the discovery of optical structure in the upper atmosphere. Sci. Adv..

[32] Gillies D.M., Donovan E., Hampton D., Liang J., Connors M., Nishimura Y., Gallardo-Lacourt B., Spanswick E. (2019). First Observations From the TREx Spectrograph: The Optical Spectrum of STEVE and the Picket Fence Phenomena. Geophys. Res. Lett..

